# Phase Equilibria in Systems Involving the Rare-Earth Oxides. Part I. Polymorphism of the Oxides of the Trivalent Rare-Earth Ions

**DOI:** 10.6028/jres.064A.030

**Published:** 1960-08-01

**Authors:** R. S. Roth, S. J. Schneider

## Abstract

The polymorphic relationships of the pure rare-earth oxides have been reinvestigated using X-ray diffraction methods for identification of phases. The oxides of the trivalent rare earth ions crystallize in three different types: A, B, and C. Each oxide has only one truly stable polymorph: La_2_O_3_, Ce_2_O_3_, Pr_2_O_3_, and Nd_2_O_3_ belong to the A type; Sm_2_O_3_, Eu_2_O_3_, and Gd_2_O_3_ to the B type; Tb_2_O_3_, Dy_2_O_3_, Ho_2_O_3_, Er_2_O_3_, Tm_2_O_3_, Yb_2_O_3_, and Lu_2_O_3_ to the C type. In addition Nd_2_O_3_, Sm_2_O_3_, Eu_2_O_3_, and Gd_2_O_3_ have low-temperature, apparently metastable, C-type polymorphs. The low-temperature form inverts irreversibly to the stable form at increasingly higher temperatures for decreasing cation radius.

## 1. Introduction

During the course of a study of phase equilibria in various rare-earth oxide systems, it was necessary to reinvestigate the polymorphic relationships of the pure rare-earth oxides. These materials had been previously investigated by Goldschmidt, Ulrich, and Barth [1]^1^ in 1925. More recently Shafer and Roy [2] have investigated some of the rare-earth oxide phases by hydrothermal means and they report some discrepancies from the original work of Goldschmidt et al.

Goldschmidt et al., [1] divided the rare-earth oxides into three main polymorphic types, A, B, and C. The oxides of the larger rare-earth cations (A type) were reported to be hexagonal and the oxides of the smallest ones (C type) cubic. The intermediate (B type) oxides had unknown symmetry. No X-ray diffraction data were given for the A- or B-type polymorphs and only unit-cell dimensions were given for the C-type cubic polymorph. The structures of the A and C types were first reported by Zachariasen [3]. Pauling [4] suggested a different structure for the A-type rare-earth oxides and this structure was confirmed by Koehler and Wollan [5] by means of neutron diffraction.

The B-type rare-earth oxide was originally observed in two different forms, reported as B_1_ and B_2_ by Goldschmidt et al. [1]. The B_2_ type was designated as the lower temperature form but was never observed as a single phase. As no X-ray data were given for either phase, it has been rather difficult for subsequent research workers to identify these phases. Douglass and Staritzky [6] grew single crystals of a polymorph of Sm_2_O_3_, which they concluded was the B type of Goldschmidt et al., and reported this modification to be monoclinic. The structure of this B-type polymorph was reported by Cromer [7].

## 2. Specimen Preparation and Test Methods

The spectrographic analyses of the oxides used in this study are listed in part II of this work [8]. The oxalates were specially purified materials supplied by E. L. Weise of the Chemistry Division of NBS. All rare-earth oxides and salts had a purity of 99.9 percent or better, with two exceptions. The samarium oxalate contained about one percent europium and, in one experiment, a neodymium oxide of about 98 percent purity was used.

For heat treatments performed in air below 1,000° C, the specimen was placed in a covered platinum crucible and heated in a commercial resistance furnace. For temperatures between 1,000° and 1,650° C an 80 percent Pt-20 percent Rh wire-wound resistance furnace was used. The specimen was contained in a platinum tube hung on fine platinum wire and was usually quenched after heating. In both furnaces the specimen was always placed immediately adjacent to the thermocouple so that the temperatures reported are probably accurate to at least ±10° C. Above 1,650° C an inductively heated iridium crucible was utilized and the temperature measured with an optical pyrometer. An arc image furnace was used in an attempt to obtain melted drops of several of the oxides.

The specimens were examined at room temperature by means of an X-ray powder diffractometer using Ni-filtered Cu radiation. The phases identified are interpreted as representing the conditions of the material at the temperature of heating. Any nonquenchable phases cannot be determined by this technique. Indexed X-ray diffraction powder patterns were used to identify the three possible polymorphs of the rare-earth oxides. The hydroxides and oxyhydroxides were identified by reference to the X-ray patterns (unindexed) listed by Schafer and Roy [2].

## 3. Experimental Data

All of the experiments performed in the present work are listed in table 1. The experiments are listed in order of increasing cation atomic number (or decreasing ionic radius), and in order of increasing temperature of heat treatment for each starting material. Continual reference to this table will be of value in clarifying the following discussion.

## 4. Comparison with Previous Results

### 4.1. La_2_O_3_

Lanthanum oxide was reported [by Goldschmidt et al., [1] to occur only in the A-type rare-earth oxide structure. However, Lohberg [9] reported preparing a C-type La_2_O_3_ by decomposing lanthanum ammonium nitrate at 450° C during a 20-hr heat treatment. This experiment was also performed by the present authors but no C-type rare-earth oxide could be identified in the X-ray pattern. Apparently the nitrate was not completely decomposed at 450° C and no rare-earth oxide structure was formed.

An attempt was also made to form the C-type La_2_O_3_ polymorph by decomposing the hydroxide at low temperatures. However, as the data in table 1 indicate, La(OH)_3_ decomposes to LaO(OH) between 200° and 300° C. This LaO(OH) is stable up to at least 500° C, too high a temperature to produce C-type La_2_O_3_. It must be concluded that considerable doubt exists as to whether pure La_2_O_3_ can be formed in the C-type polymorph. This question cannot be answered unless a method is found to produce La_2_O_3_ by decomposing a lanthanum compound below 500° C.

### 4.2. Ce_2_O_3_ and Pr_2_O_3_

Goldschmidt et al. [1] have suggested that B-type Ce_2_O_3_ might be obtained by a heat treatment of several days duration in a stream of hydrogen at about 600° C. They report the A-type of Ce_2_O_3_, obtained by heating ceric oxide in a stream of hydrogen at 1,250° C for 30 min. No attempt has been made to heat cerium oxide in hydrogen in the present work. One attempt to form Ce_2_O_3_ from cerous oxalate at 500° C in vacuum (10^−4^ mm Hg) was unsuccessful, resulting in CeO_2_. Data on Nd_2_O_3_ (see sec. 4.3.) indicate that it is very unlikely that Ce_2_O_3_ could form the B structure. However, it may be possible that the C type can form at low temperatures.

Praseodymium oxide was formed in the A type in the present work by heating Pr_6_O_11_ in vacuum (10^−5^ mm) at 1,010° C. As in the case of Ce_2_O_3_ it seems unlikely that the B type would exist. However, the C type may very possibly form at low temperatures.

### 4.3. Nd_2_O_3_

Neodymium oxide was found by Goldschmidt et al. [1] to be stable in the B type over a large temperature range, transforming to the A type at about 1,000° C, although it could be found metastably in short runs as high as 1,300° C. The C modification of Nd_2_O_3_ was first described by Lohberg [9], who obtained this structure type at 700° and 875° C and found that it transformed directly to the A type at about 940° C. Lohberg was unable to prepare Nd_2_O_3_ in the B modification.

Shafer and Boy [2] have studied the stability of the phases in some rare-earth oxide—water systems by hydrothermal techniques. They report B-type Nd_2_O_3_ to be stable at intermediate temperatures and pressures (for instance 950° C—5,000 lb/in.^2^).

In the present work, Nd_2_O_3_ was found to transform from the C type directly to the A type at atmospheric pressure and approximately 650° C. Attempts to form the B-type oxide by reproducing the experiments of Goldschmidt et al. [1] and Shafer and Roy [2] resulted in either C- or A-type Nd_2_O_3_ or else Nd(OH)_3_ or NdO(OH).

Shafer and Roy report an X-ray powder diffraction pattern for B-type Nd_2_O_3_. This work was performed before the publication of the monoclinic parameters of B-type Sm_2_O_3_ by Douglass and Staritzky [6] and the B-Nd_2_O_3_ pattern was not indexed. However, a comparison of Shafer and Roy’s pattern with those of the known B-type oxides indicates that it does have the B structure. Therefore, it must be concluded that the B polymorph is stable in the range indicated by Shafer and Roy. The reason it could not be synthesized in the present work probably depends on the rate of reaction of this phase with H_2_O. Shafer and Roy were probably able to obtain the B-type Nd_2_O_3_ by allowing the steam to escape first and then temperature quenching the dry specimen. With the equipment available for the present work, this was not possible and the specimen was in contact with hot water for several minutes during cooling. A specimen of B-type Nd_2_O_3_ prepared by Shafer and Roy in their original work was sent to the present authors. However it was found that the specimen now contained only NdO(OH), probably having hydrated over the years at atmospheric pressure. In compositions reported in part II of this study [8], it was observed that B-type solid solutions containing 90 percent or more of Nd_2_O_3_ had a much stronger tendency to hydrate than did the A or C modifications of Nd_2_O_3_.

It may be concluded, therefore, that pure Nd_2_O_3_ at atmospheric pressure will be formed only in the C and A polymorphic types. However under hydrothermal conditions the B type may occur at intermediate temperatures. Whether the B type could occur in pure Nd_2_O_3_-H_2_O mixtures in a closed system cannot be determined at the present time as the water cannot be driven out fast enough in such a system to prevent hydration. As the B-type Nd_2_O_3_ does not occur, under atmospheric conditions, it seems unlikely that the B type would form at all with Pr_2_O_3_ or Ce_2_O_3_.

### 4.4. Sm_2_O_3_

Goldschmidt et al., report both the C and B modifications for Sm_2_O_3_. They state that type C will not transform to B with heat treatments of several days duration at 800° C, 700° C, or 600° C. However, they state that a mixture of B and C types was formed by heating the hydroxide as low as 735° C. In the present work the C polymorphic type formed from samarium oxalate did not transform to the B structure with 1 to 2 weeks heat treatment at several temperatures up to 900° C. A sample of C-Sm_2_O_3_ whose diffuse powder diffraction lines indicated poor crystallinity readily formed the B type at 1,000° C in a few hours.

It appears that the C–B transformation in Sm_2_O_3_ is a rate process depending on the crystallinity of the C type. Poorly crystallized C-type material quickly transforms to the B type at relatively low temperatures whereas the more crystalline C-type samples will be transformed only with greater difficulty.

Goldschmidt et al. were unable to transform the B-Sm_2_O_3_ back to the C type even with many days annealing. They concluded that with Sm_2_O_3_ the C–B transformation may be monotropic. The fact that the C–B transformation temperature apparently depends on the crystallinity of the starting material is another indication of the monotropic nature of the reaction.

Goldschmidt et al. suggested that, for Sm_2_O_3_, the stable phase was type A at the melting point and this quickly transformed to B on cooling. A quickly cooled drop of Sm_2_O_3_, melted in an arc-image furnace in the present work, showed X-ray powder diffraction lines of the B type only. It therefore seems unlikely that pure Sm_2_O_3_ can be quenched in the A-structure, if such a structure exists. Only high temperature X-ray measurements at approximately 2,300° C could answer the question of the occurrence of the A structure.

### 4.5. Eu_2_O_3_

Europium oxide was obtained as the C type by Goldschmidt et al., at 735° C and 750° C, and as the B type at 1,100° C. Curtis and Tharp [10] reported the C–B transformation temperature for Eu_2_O_3_ as approximately 1,050° C. In the present work the C modification of Eu_2_O_3_ did not transform to B even after 114.5 hr at 1,050° C. The C–B transformation in Eu_2_O_3_ may also depend on crystallinity. However, by combining the data from the present experiments with those of Curtis and Tharp [10] the transformation temperature can be concluded to be about 1,075° C.

### 4.6. Gd_2_O_3_

Goldschmidt et al. [1] found the C-type Gd_2_O_3_ to be stable at 600° and at 750° C. However, their X-ray powder pattern of gadolinium oxide which had been heated to 800° to 900° C showed strongly the lines of a crystalline phase which apparently had relatively high symmetry and was called B_2_ type oxide. Crystal type B_1_ was observed by Goldschmidt et al. in a specimen heated at 1,300° C.

Guentert and Mozzi [11] reported high temperature X-ray diffraction experiments on Gd_2_O_3_. The monoclinic form, identified with Goldschmidt’s B_1_, did not occur at 1,200° C but was partially formed at 1,400° C. They concluded that their results indicated a direct transition from the cubic to the monoclinic form, without any evidence for the existence of the B_2_ type.

In the present work the C–B transformation in Gd_2_O_3_ was observed in heatings of several days to take place at approximately 1,250° C. Again no evidence of the B_2_ type was found in experiments involving the C–B transformation. Dr. T. F. W. Barth [12] has suggested, in a private communication, that “Perhaps the B_2_ phase simply is caused by impurities.” The present authors were unable to obtain the data for the *d* values which were identified by Goldschmidt et al., as being characteristic of the B_2_ phase. Therefore the B_2_ phase cannot be definitely explained.

**Figure 1:**
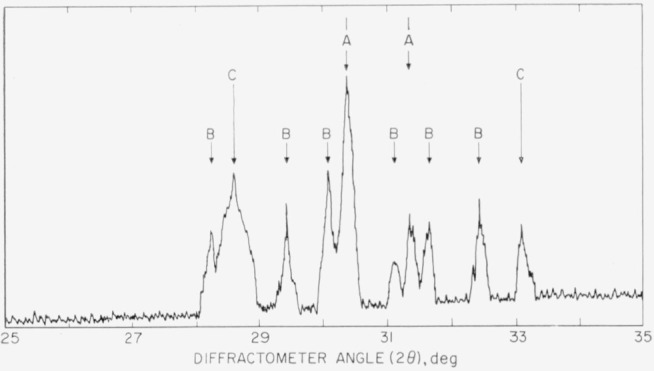
Portion, of an X-ray diffraction powder pattern from the surface of a pellet of *Gd_2_O_3_* heated al 1,200° C for 2 weeks, showing a mixture of all three crystal types A, B, and C. Ni-filtered Cu radiation.

As the C–B transformation was not found to be reversible in Sm_2_O_3_ it was thought that the reversibility of this transformation in Gd_2_O_3_ should be checked. A pellet of the B-type Gd_2_O_3_ was heated just below the transformation temperature at 1,200° C for 2 weeks. The body of the pellet was found to contain only the B-crystal type. However, the surface of the pellet showed both C and A polymorphic types in addition to the B-type (fig. 1). As the (002) peak was much stronger than the (101), it appears the A-Gd_2_O_3_ was highly oriented on the surface of the pellet. From this data the present authors conclude that A-Gd_2_O_3_ is only a metastable phase and that the C–B transformation is not truly reversible.

### 4.7. Tb_2_O_3_

In the present study, Tb_4_O_7_ was converted to a phase having the C-type crystal structure by heating in argon at 1,500° C. However it is possible that this reaction did not proceed completely to Tb_2_O_3_ and may represent a solid solution of Tb_2_O_3_ with excess oxygen.

### 4.8. Dy_2_O_3_, Ho_2_O_3_, Y_2_O_3_

These three oxides have been found to occur only in the C-type modification. However, Goldschmidt et al. state that the B_1_–C transformation of dysprosium oxide appears to proceed spontaneously. This was based on the observation that: “After heating at the platinum melting point, dysprosium oxide showed modification C, possibly pseudomorphic after B.”

In the present work, a bar of Dy_2_O_3_ was heated in an arc image furnace and very quickly cooled. Due to the thermal shock characteristics of Dy_2_O_3_ the hot portion of the bar broke up with explosive force, so that it could not be determined whether melting had occurred. However, modification C was the only type identified in the X-ray pattern. It may be concluded that pure Dy_2_O_3_ can form only the C-type structure, although very small amounts of impurities may possibly enable a B type to become stable. Both Ho_2_O_3_ and Y_2_O_3_ have been found to crystallize only in the C type.

### 4.9. Er_2_O_3_, Tm_2_O_3_, Yb_2_O_3_, and Lu_2_O_3_

These rare earth oxides have been found only in the C type. Goldschmidt et al. examined Yb_2_O_3_ at low temperatures to see if a further type D would appear, with negative results. There is no evidence to suggest any other modification of the pure rare-earth oxides and the present work (table 1) is not in disagreement with the conclusion that the C-type is the only modification to appear in the oxides of the smaller rare-earth ions.

## 5. General Conclusions

### 5.1. Stability of the Polymorphic Types

**Figure 2:**
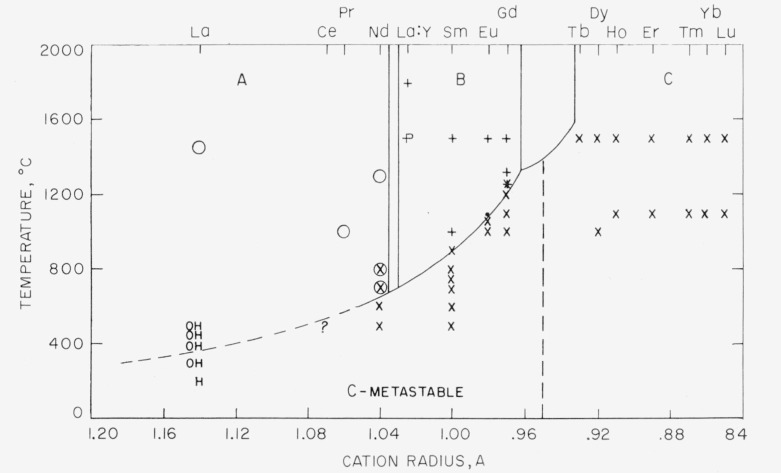
Stability relations of the A, B, and C polymorphic forms of the rare-earth sesquioxides. *O*, A type; +, B type; X, C type; P, perovskite; La:Y, 1:1 La_2_O_3_:Y_2_O_3_; H, hydroxide; OH, oxyhydroxide; ?, structure and oxidation state unknown.

The stability relations of the polymorphs encountered in this study are summarized in figure 2. In this figure the temperature has been plotted on the ordinate and the radii of the rare-earth ions (instead of the atomic number as was done by Goldschmidt) have been plotted on the abscissa. The radii used here are those given by Ahrens [13]. This diagram shows the A-type rare-earth oxide structure to be stable for the larger ions, the B type for the intermediate ions, and the C type for the smaller ions. In addition, the C-type structure is shown to occur metastably at low temperatures for all the A- and B-type oxides except La_2_O_3_. This oxide could not be formed by decomposition at low enough temperatures to confirm the existence of the C type. The A–B and B–C boundaries are shown as two-phase areas to correspond with the diagrams drawn for part II of this study [8] which show the relationships of these polymorphs in all possible binary and ternary combinations. The upper stability temperature of the metastable C field, for any given oxide, probably depends upon the starting material. The C–B reaction has not been reversed experimentally and is probably monotropic.

Goldschmidt et al. [1] state that the three crystal types A, B, and C show “succeeding thermal relations,” A being stable at the highest temperatures, B at intermediate temperatures, and C at the lowest temperatures. This conclusion cannot be drawn from the present study. Rather, the three crystal types show relations only to the cation size, A for the largest ions, B for the intermediate ions, and C for the smaller ions. As the low→High reaction of the C polymorph to either B or A is probably monotropic, the C polymorph should not be considered as a stable phase for these oxides.

During the interval between the publication of Goldschmidt’s work [1] and the indexing of the X-ray powder pattern of the B type by Douglass and Staritzky [6], the B polymorph was almost totally ignored and the rare-earth oxides were generally classified into two types, A and C. There is no longer any excuse for this binary classification. As has been shown by the present work, as well as that of Goldschmidt et al. [1], Shafer and Roy [2], and others, the trivalent rare-earth oxides must be classified into the three groups A, B, and C with the B type recognized as a stable crystal phase. The B polymorph is more stable in impure materials than it is in the pure rare earths, as is shown for various binary and ternary systems studied by the present authors [8]. This stability of the B type in impure systems may account for the report of a B polymorph in the oxides of the larger rare-earth ions such as Nd_2_O_3_.

Goldschmidt, Ulrich, and Barth have given the impression that the three crystal types in the rare-earth oxides, in general, showed enantiotropic relations. However, no enantiotropic reactions were found in the present work. The C–B transformation occurs readily enough. However, the B–C transformation was not obtained except as a surface reaction in Gd_2_O_3_ involving the formation of the A polymorph as well. Goldschmidt et al. claimed this reaction was enantiotropic on the basis of a spontaneous B–C reaction in dysprosium oxide. The present work shows no indication of a B type for Dy_2_O_3_.

No A–B or B–A reaction was found in the present work. Goldschmidt observed this reaction to proceed in both directions with Nd_2_O_3_. As it seems that B-Nd_2_O_3_ can exist in the pure oxide only under hydrothermal conditions the reversibility of this reaction cannot be checked. The A–B transformation, which was reported by Goldschmidt et al., to take place spontaneously in Sm_2_O_3_, also was not observed.

The transformation C–A observed in the present work for Nd_2_O_3_ was not observed at all by Goldschmidt. The A-type Nd_2_O_3_ cannot be converted to C-type Nd_2_O_3_ without going through an intermediate hydrated state.

Shafer and Roy [2] have concluded that the inversions in the rare-earth oxides “appear to be genuine reversible polymorphic transitions.” This conclusion appears to be based on an experiment in which B-type Nd_2_O_3_ was transformed to C-type Nd_2_O_3_ hydro thermally. However it must be realized that in the process of heating this specimen the B-Nd_2_O_3_ was necessarily first transformed to a hydrated crystalline type, so that the reversibility of the direct transformation is left open to question. It may be concluded therefore that all the polymorphic reactions of the trivalent rare-earth oxides are irreversible and probably monotropic.

### 5.2. Unit-Cell Parameters and Radii of the Trivalent Rare-Earth Cations

The unit-cell dimensions of the various polymorphs of the rare-earth oxides are listed in table 2. All of these values are derived from data obtained in the present study with the exception of the B-type Nd_2_O_3_, which was calculated from the X-ray pattern listed by Shafer and Roy [2].

**Figure 3:**
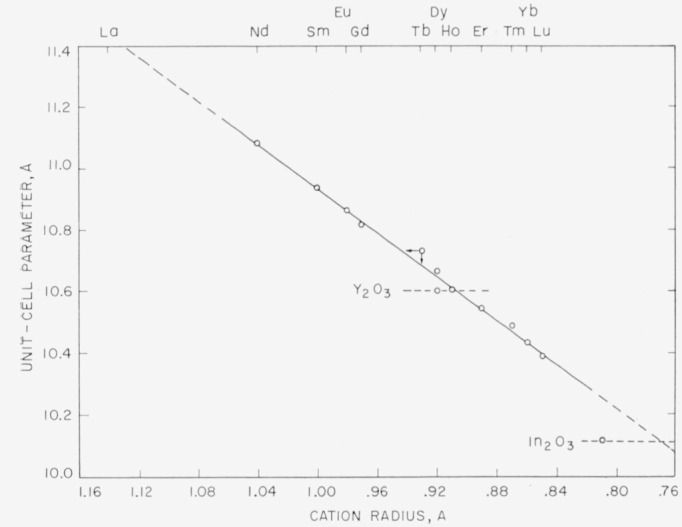
Linear relationship between the unit-cell dimensions and the cation radius for the C-type cubic rare-earth oxide structures.

The unit-cell dimensions of the C-type cubic polymorphs are plotted in figure 3 against the radii of the cations, as listed by Ahrens [13]. It can be seen that the unit cell dimensions of the oxides of the rare-earth ions from Nd^+3^ to Lu^+3^ fall on a straight line. The straight line relationship is required for the C-type polymorphs, as Ahrens’ radii were originally calculated from unit-cell dimensions of the C-type oxides. The fit to a straight line would be more exact if the radii were given to three decimal points as suggested by Templeton and Dauben [14]. The Tb_2_O_3_ parameter is the only one significantly off the straight line as indicated by the arrows. Either the radius of Tb^+3^ listed by Ahrens is too small or else the reduction of the present specimen was not complete. It should be pointed out that an extension of this straight line to the radius of La^+3^ predicts the unit cell dimension of C-type La_2_O_3_ close to the value of 11.4 A reported by Lohberg [9].

The values of the unit-cell dimensions of Y_2_O_3_ and In_2_O_3_ do not fall on this line when the radii given by Ahrens for Y^+3^ and In^+3^ are utilized. It must be concluded that both Y^+3^ and In^+3^ have slightly different effective radii when occurring in oxides of the C-type structure, and probably also in other oxide structure types. The radius of Y^+3^ is found to be just slightly smaller than that of Ho^+3^, 0.91 A. The extension of the straight line intersects the unitcell dimension of In_2_O_3_ at 0.77 A rather than the value of 0.81 A given by Ahrens. Using the unit-cell parameters of Sc_2_O_3_ (9.81 A) and Mn_2_O_3_ (9.43A) listed by Donnay and Nowacki [15] the effective radii of Sc^+3^ and Mn^+3^ in rare-earth-oxide-type compounds are found to be approximately 0.68 A and 0.56 A, respectively.

Templeton and Dauben [14] have calculated ionic radii of the rare-earth ions using a value of 0.21441 *a* (for the C-type rare-earth oxide structure) for the cation-oxygen distance. They emphasize that the relative difference between any two trivalent rare-earth cations can be found much more accurately than the absolute radii. For this reason the more generally accepted values of Ahrens, as modified by figure 3, are used for the present work.

**Figure 4:**
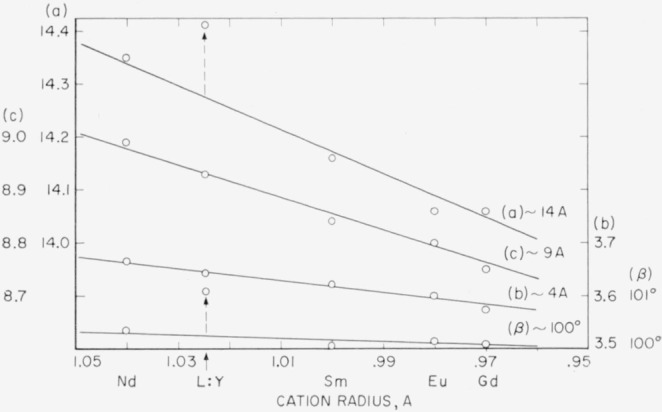
Linear relationship between the unit-cell dimensions and the cation radius for the B-type monoclinic rare-earth oxide structures.

In figure 4 the unit-cell parameters of the B-type oxides are plotted against the ionic radii. The a, *b*, and *c* parameters all increase significantly with increasing cation radius. The change in *β* is almost negligible. In addition to the unit cell dimensions of Nd_2_O_3_, Sm_2_O_3_, Eu_2_O_3_, and Gd_2_O_3_, the dimensions of the B-type phase reported to occur at 1:1 La_2_O_3_: Y_2_O_3_^2^ [16] are plotted using an average radius for the two cations. It can be seen that the *a* and *β* parameters for 1:1 La_2_O_3_:Y_2_O_3_ occur at very significantly larger values than would be expected from the plot of the pure oxides. It will be shown in part II [8] that many solid solutions of the B type have this different relationship.

**Figure 5:**
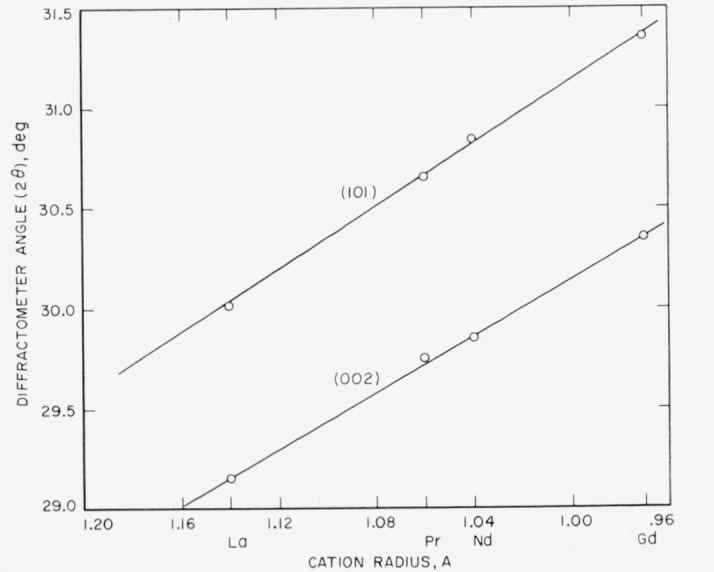
Linear relationship between the diffraction angle 2θ and cation radius for the (002) and (101) peaks of the A-type hexagonal rare-earth oxide structures.

It can be inferred from figure 5 that the unit-cell dimensions of the A-type rare-earth oxides also change linearly with change in the radius of the cation. It may be seen that the (002) and (101) values observed for the metastable A-type Gd_2_O_3_ fall on the same straight lines as those drawn through the values for the stable A-type oxides.

### 6. Summary

The polymorphic forms of the oxides of the trivalent rare-earth ions have been reinvestigated. The overall picture of polymorphism in trivalent rare-earth oxides is slightly changed as compared with the original study of Goldschmidt et al. [1]. Lanthanum oxide has been found to occur only in the A-type hexagonal structure. Neodymium oxide forms the C-type cubic structure at low temperatures and inverts irreversibly to the A-type at about 650° C. The B-type monoclinic structure reported for Nd_2_O_3_ was not encountered in the present work. Samarium oxide, Eu_2_O_3_, and Gd_2_O_3_ crystallize in the C-type at low temperatures and invert directly and irreversibly to the B-type monoclinic structure at about 950°, 1,075°, and 1,225° C, respectively. TheA-type structure was not found to occur at high temperatures in Sm_2_O_3_ but was found to occur metastably in Gd_2_O_3_ as a surface reaction when the B type was held at 1,200° C for several weeks. All the rare-earth oxides which have cations smaller than Gd^+3^ showed only the C-type cubic modification.

The transformation of the low-temperature C-type structure to either A or B type was concluded to be a rate process, depending on the degree of crystallinity of the C polymorph. This transformation appears to be irreversible, probably monotropic. No enantiotropic transformations were encountered in this study.

The unit-cell dimensions of the oxides of all three types are shown to vary linearly with the radius of the cation. The linear relationship holds true regardless of whether the polymorph is stable or metastable.

## Figures and Tables

**Table 1:** Experimental data on the polymorphic relations of the rare-earth oxides

Starting material	Heat treatment^1^		Pressure 2	Phases identified by X-ray powder diffraction	Remarks
	Temp.	Time			
					
	° *C*	*hr*			
La(OH)_3_^3^	200	144	…………………	La (OH)_3_	
Do	300	144	…………………	LaO(OH)+La(OH)_3_	Very poor X-ray pattern.
Do	350	168	…………………	LaO(OH)	
Do	400	168	…………………	do	
Do	450	168	…………………	do	
Do	500	168	…………………	do	
La(NO_3_)·2NH_4_NO_3_·4H_2_O	450	20	…………………	Unidentified	Does not contain either C- or A-type rare-earth oxide.
Do	450	192	…………………	do	
La(NO_3_)·6H_2_O	450	20	…………………	do	Not A- or C-type rare-earth oxide.
Ce_2_(C_2_O_4_)_3_·10H_2_O	500	96	10^−4^ mm Hg.	CeO_2_	Cerous oxalate must be heated in hydrogen to maintain trivalent state.
Pr_6_O_11_	1,010	.83	10^−5^ mm Hg	A-type Pr_2_O_3_	
Nd(C_2_O_4_)_3_·10H_2_O	500	40	…………………	C-type Nd_2_O_3_	
Do	500	168	…………………	4Unidentified+ C-type Nd_2_O_3_	
Do	600	120	…………………	C-type Nd_2_O_3_	
Do	700	144	…………………	C-type Nd_2_O_3_+A-type Nd_2_O_3_	No B-type Nd_2_O_3_.
Do	800	144	…………………	A-type Nd_2_O_3_+C-type Nd_2_O_3_	Do
Nd(C_2_O_4_)_3_·10H_2_O	1,300	.083	…………………	A-type Nd_2_O_3_	
Nd(C_2_O_4_)_3_·10H_2_O^5^	600	168	20,000 psi	Nd(OH)_3_	Sealed in Pt tube with H_2_O added.
Do.^5^	600	168	20,000 psi	NdO(OH)	Sealed in Pt tube without adding H_2_O.
Do.^5^	950	42	5,000 psi	do	
Do.^6^	950	42	5,000 psi	do	Sealed in Pt tube with H_2_O.
Do.^6^	950	42	5,000 psi	A-type Nd_2_O_3_	Sealed in Pt tube without adding H_2_O.
Nd(OH)_3_^7^	600	16	…………………	C-type Nd_2_O_3_	
Do	950	48	5,000 psi	NdO(OH)+Nd(OH)_3_	Heated in unsealed Pt tube.
Do.^5^	950	48	5,000 psi	NdO(OH)+A-type Nd_2_O_3_	Sealed in Pt tube without adding H_2_O.
Do	600	288	…………………	C-type Nd_2_O_3_	
Sm_2_O_3_	1,000	2	…………………	B-type Sm_2_O_3_	
Do	1,500	1.5	…………………	do	
Sm_2_O_3_	(9)	………….	…………………	C-type Sm_2_O_3_?	Very poorly crystalline.
Do	500	4	…………………	C type + B-type Sm_2_O_3_	
Do	500	168	…………………	do	B-type probably present in “as received” material.
Sm_2_(C_2_O_4_)_3_·10H_2_O	500	336	…………………	C-type Sm_2_O_3_	
Do	600	168	…………………	do	
Do	700	192	…………………	do	
Do	750	216	…………………	do	
Do	800	192	…………………	do	
Do	900	168	…………………	do	
Sm_2_O_3_^5^	600	168	20,000 psi	SmO(OH)	Sealed in Pt tube with H_2_O.
Do.^5^	600	168	20,000 psi	C-type Sm_2_O_3_+SmO(OH)	Sealed in Pt tube without adding H_2_0.
Do.^5^	950	6	5,000 psi	B-type Sm_2_O_3_+SmO(OH)	Do.
Sm_2_O_3_	(10)	(10)	…………………	B-type Sm_2_O_3_	Bar previously calcined at 1475° C for 15 hr.
Eu_2_O_3_	1,000	2	…………………	C-type Eu_2_O_3_	
Do	1,500	1.5	…………………	B-type Eu_2_O_3_	
Eu_2_O_3_	1,050	114.5	…………………	C-type Eu_2_O_3_	
Gd_2_O_3_	1,000	2	…………………	C-type Gd_2_O_3_	
Do	1,500	1.5	…………………	B-type Gd_2_O_3_	
Gd_2_O_3_	1,000	68	…………………	C-Type Gd_2_O_3_	
Do	1,100	42	…………………	do	
Do	1,200	42	…………………	do	
Do	1,250	72	…………………	C-Type+B-type Gd_2_O_3_	No sign of B_2_-type Gd_2_O_3_.
Do	1,319	64	…………………	B-type Gd_2_O_3_	
Do	1,200	336	…………………	B-type Gd_2_O_3_+C-type Gd_2_O_3_+A-type Gd_2_O_3_	C- and A- Gd_2_O_3_ only surface effect.
Gd_2_O_3_	600	168	20,000 psi	GdO(OH)	Sealed in Pt tube with H_2_O added.
Do	600	168	20,000 psi	C-type Gd_2_O_3_	Sealed in Pt tube without adding H_2_O.
Do	950	6	5,000 psi	do	Do.
Gd(OH)_3_^8^	850	.167	…………………	C-type Gd_2_O_3_	
Tb_4_O_7_	1,500	1.5	…………………	2 cubic phases (?)	
Do	1,500	0.33	…………………	C-type Tb_2_O_3_	Heated in argon atmosphere.
Dy_2_O_3_	1,000	2	…………………	C-type Dy_2_O_3_	
Do	1,500	1.5	…………………	do	Bar previously calcined at 1475° C 15 hr.
Dy_2_O_3_	(11)	(11)	…………………	do	
Ho_2_O_3_	1,200	6	…………………	C-type Ho_2_O_3_	
Do	1,500	6	…………………	do	
Er_2_O_3_	1,200	6	…………………	C-type Er_2_O_3_	
Do	1,500	6	…………………	do	
Tm_2_O_3_	1,100	4	…………………	C-type Tm_2_O_3_	
Do	1,500	6	…………………	do	
Yb_2_O_3_	1,100	4	…………………	C-type Yb_2_O_3_	
Do	1,500	6	…………………	do	
Lu_2_O_3_	1,100	4	…………………	C-type Lu_2_O_3_	
Do	1,500	6	…………………	do	
Y_2_O_3_	1,350	10	…………………	C-type Y_2_O_3_	
Do	2,000	0.25	…………………	do	

1 The heat treatment for each specimen includes all previously listed lower temperature heatings for the same starting material.

2 All specimens were treated at atmospheric pressure except where indicated in this column.

3 A-type La_2_O_3_ boiled in distilled H_2_O for 2 hr; dried overnight at 110° C; X-ray powder diffraction pattern shows only La(OH)3.

4 Unidentified phase probably due to reaction with atmosphere after removal from furnace and before subjecting to X-ray diffraction. This was the only specimen in the series to wait several days between heat treatment and X-ray examination.

5 Calcined at 600° C in Pt crucible, but probably partially rehydrated before being sealed in Pt tube.

6 Calcined at 600° C in Pt crucible, recalcined at 600° C overnight in Pt tube and sealed.

7 Impure A-type Nd_2_O_3_ boiled in distilled H_2_O for 2 hr, dried overnight at 110° C. X-ray powder diffraction patterns show only Nd(OH)_3_.

8 Gd(OH)3 formed by dissolving Gd_2_O_3_ in HCl and precipitating with (NH_3_)OH, precipitate washed and dried under infrared lamp.

9 As received.

10 Melted drop in arc image furnace.

11 Melted(?) drop in arc image furnace.

**Table 2:** Unit-cell dimensions of the rare-earth oxides

Oxide	Radius of cation^1^	A type		B type				C type	
		a	c	a	b	c	*β*	a	a, least squares^2^
									
		*A*	*A*	*A*	*A*	*A*		*A*	*A*
La_2_O_3_	1.14	3.93	6.12	……………	……………	……………	……………	……………	……………
Ce_2_O_3_	1.07	……………	……………	……………	……………	……………	……………	……………	……………
Pr_2_O_3_	1.06	3.85	6.00	……………	……………	……………	……………	……………	……………
Nd_2_O_3_	1.04	3.82	5.98	14.35	3.666	8.99	100.34°	11.080	……………
Sm_2_O_3_	1.00	……………	……………	14.16	3.621	8.84	100.05°	10.934	……………
Eu_2_O_3_	0.98	……………	……………	14.06	3.601	8.80	100.15°	10.860	……………
Od_2_O_3_	.97	3.76	5.89	14.06	3.572	8.75	100.10°		10.8122
Tb_2_O_3_	.93	……………	……………	……………	……………	……………	……………	10.729	……………
Dy_2_O_3_	.92	……………	……………	……………	……………	……………	……………	……………	10.6647
Ho_2_O_3_	.91	……………	……………	……………	……………	……………	……………	……………	10.6065
Y_2_O_3_	3(.91)	……………	……………	……………	……………	……………	……………	……………	10.6021
Er_2_O_3_	.89	……………	……………	……………	……………	……………	……………	……………	10.5473
Tm_2_O_3_	.87	……………	……………	……………	……………	……………	……………	……………	10.4866
Yb_2_O_3_	.86	……………	……………	……………	……………	……………	……………	……………	10.4334
Lu_2_O_3_	.85	……………	……………	……………	……………	……………	……………	……………	10.3907
In_2_O_3_	3(.77)	……………	……………	……………	……………	……………	……………	……………	10.1178

1 Radii of the cations taken from Ahrens [13] except where noted.

2 The data were fitted by least squares by Cohen’s extrapolation method as discussed by Azároff and Buerger, The Powder Method. (McGraw-Hill Hook Co., Inc., New York, N.Y., 1958).

3 These are the radii derived from the unit-cell dimensions in the present study.

## Footnotes

1 Figures in brackets indicate the literature references at the end of this paper.

2 The unit-cell parameters of the B-type 1:1 La_2_O_3_:Y_2_O_3_, previously heated at 1,800° C for 1 hr, are: *a* = 14.41 A, *b* = 3.643 A, *c* = 8.93 A, *β* = 101.09°.
